# Deep Venous Thrombosis Within Two Weeks of Initiating Oral Semaglutide (Rybelsus): A Case Report

**DOI:** 10.7759/cureus.100836

**Published:** 2026-01-05

**Authors:** Maamoun Alsermani, Baraa Ali Alkahmous, Arwa A Alhutayrashi, Fai S Alshalhoob, Sarah abulrahman Almosaiteer, Eman Aljubayri, Assia Saleh Alwabel, Anas Sermani

**Affiliations:** 1 Hematology, Prince Mohammed Bin Abdulaziz Hospital, Riyadh, SAU; 2 Medicine and Surgery, Cluster 2 Riyadh – Ministry of Health, Riyadh, SAU; 3 Medicine, Princess Nourah bint Abdulrahman University, Riyadh, SAU; 4 Internal Medicine, Cluster 2 Riyadh – Ministry of Health, Riyadh, SAU; 5 Internal Medicine, Ankara Yıldırım Beyazıt University, Ankara, TUR

**Keywords:** glp-1 receptor agonist, pharmacovigilance, rybelsus, semaglutide, venous thromboembolism

## Abstract

Glucagon-like peptide-1 receptor agonists (GLP-1RAs) are increasingly prescribed for type 2 diabetes mellitus and weight management, with growing off-label use in young adults. While GLP-1RAs are generally well tolerated, emerging evidence suggests potential associations with thromboembolic events.

We report the case of a 16-year-old male with mild conventional risk factors for thrombosis [overweight body mass index (BMI) and prolonged sedentary behavior] who developed acute left lower extremity deep venous thrombosis (DVT) within two weeks of initiating oral semaglutide (Rybelsus, 7 mg daily) for weight reduction without medical supervision. He presented with progressive leg pain and swelling, and duplex ultrasonography revealed thrombosis of the distal superficial femoral and popliteal veins. An extensive thrombophilia workup, including cardiolipin and β2-glycoprotein antibodies, returned negative. Anticoagulation with low molecular weight heparin followed by apixaban resulted in significant clinical improvement.

This case suggests a possible association between oral semaglutide and venous thrombosis in an adolescent, highlighting the need for careful supervision during off-label use. Possible contributing mechanisms include hemoconcentration from gastrointestinal adverse effects, interaction with sedentary behavior, and potential effects of GLP-1RAs on coagulation or endothelial function. Similar cases have been described with injectable semaglutide and other GLP-1RAs, but limb DVT associated with oral semaglutide has not previously been reported.

Oral semaglutide may rarely predispose to thrombotic complications. Clinicians should maintain vigilance, especially in adolescents and individuals self-prescribing for weight loss. Further pharmacovigilance and mechanistic studies are warranted to clarify this potential association.

## Introduction

Semaglutide is a glucagon-like peptide-1 receptor agonist (GLP-1RA) approved for the treatment of type 2 diabetes mellitus and, more recently, for obesity management. It is available both as a once-weekly subcutaneous injection and a once-daily oral tablet (Rybelsus). The cardiovascular and renal benefits of semaglutide have been demonstrated in large randomized controlled trials, including the SUSTAIN and PIONEER programs, establishing it as a preferred therapeutic option in diabetes care [1,2].

The most common adverse events associated with semaglutide are gastrointestinal, including nausea, vomiting, and diarrhea, which are usually self-limiting and dose dependent [3]. However, concerns have been raised regarding its association with venous thromboembolism (VTE), including pulmonary embolism, portal vein thrombosis, and atypical site thrombosis [4-6]. The mechanisms may involve hemoconcentration from dehydration or hypercoagulability secondary to gastrointestinal effects.

Here, we describe a case of limb deep venous thrombosis (DVT) developing within two weeks of initiating oral semaglutide in a 16-year-old adolescent. To our knowledge, this is the first reported case with the oral formulation. The patient had mild conventional risk factors [overweight body mass index (BMI) and sedentary behavior] and used the medication off-label without medical supervision.

## Case presentation

A 16-year-old young man with a history of iron deficiency anemia on oral iron supplementation presented to the emergency department with progressive pain and swelling of the left lower limb for the past three days. He reported no history of trauma or any injury to the lower limb, denied any recent surgical procedures, prolonged travel, or immobility, and had no personal or family history of DVT. Upon systemic review, he denied any symptoms suggestive of connective tissue disease, such as rash, fever, arthralgia, or nail changes. He also reported no personal or family history of malignancy, unintentional weight loss, or lymphadenopathy

The patient started oral semaglutide (Rybelsus, 7 mg daily) without a prescription for weight loss. Over the two weeks, he reported vomiting one to two times per week. Appetite decreased minimally, and oral intake was slightly reduced. No diarrhea or significant weight loss occurred. He reported prolonged sedentary behavior, sitting seven to eight hours daily, with minimal physical activity. His lifestyle included high-processed food intake.

On examination, he was afebrile and hemodynamically stable, with a BMI of 26.3 kg/m². Cardiovascular, respiratory, and abdominal examinations were unremarkable. The left lower limb was swollen compared to the right, with tenderness over the calf and popliteal region, increased warmth, but no erythema. Peripheral pulses were intact. Laboratory investigations revealed microcytic hypochromic anemia and mild neutrophilic leukocytosis, with otherwise normal renal, hepatic, and thyroid function tests (Table 1).

**Table 1:** Laboratory investigation ESR: erythrocyte sedimentation rate; CRP: c‑reactive protein; LDL cholesterol: low‑density lipoprotein cholesterol; HbA1c: hemoglobin A1c (glycated hemoglobin); TIBC: total iron binding capacity; TSH: thyroid stimulating hormone

Laboratory test	Value	Normal range
Sodium	136 mmol/L	136–146 mmol/L
Potassium	4.3 mmol/L	3.6–5.1 mmol/L
Chloride	108 mmol/L	98–107 mmol/L
Creatinine	64 µmol/L	45–84 µmol/L
Urea	2.7 mmol/L	2.67–8.07 mmol/L
Albumin	38 g/L	>21 g/L
Calcium	2.21 mmol/L	2.23–2.58 mmol/L
Amylase	37 units/mL	28–100 units/mL
Lipase	45 IU/L	13–60 IU/L
Aspartate aminotransferase	23 IU/L	<32 IU/L
Alanine aminotransferase	26 IU/L	<33 IU/L
Hemoglobin	10.9 g/dL	117–155 g/L
Mean corpuscular volume	60 fL	81–100 fL
Platelets	282 ×10⁹/L	150–450 x10^9/L
ESR	22 mm/hr	
CRP	4.93 mg/dL	0.01–0.50 mg/dL
Triglyceride	0.71 mmol/L	0.00–2.26 mmol/L
LDL cholesterol	2.1580 mmol/L	<3.8000 mmol/L
HbA1c	6.6 %	4–6 %
Ferritin	25.2 ng/mL	21.8–274.6 ng/mL
Iron	3 µmol/L	11.6–31.3 µmol/L
TIBC	64.10 µmol/L	
B12	234 pmol/L	139–651 pmol/L
Folate	28.3 nmol/L	7.9–46.4 nmol/L
TSH	1.29 mIU/L	0.35–4.94 mIU/L

**Figure 1:**
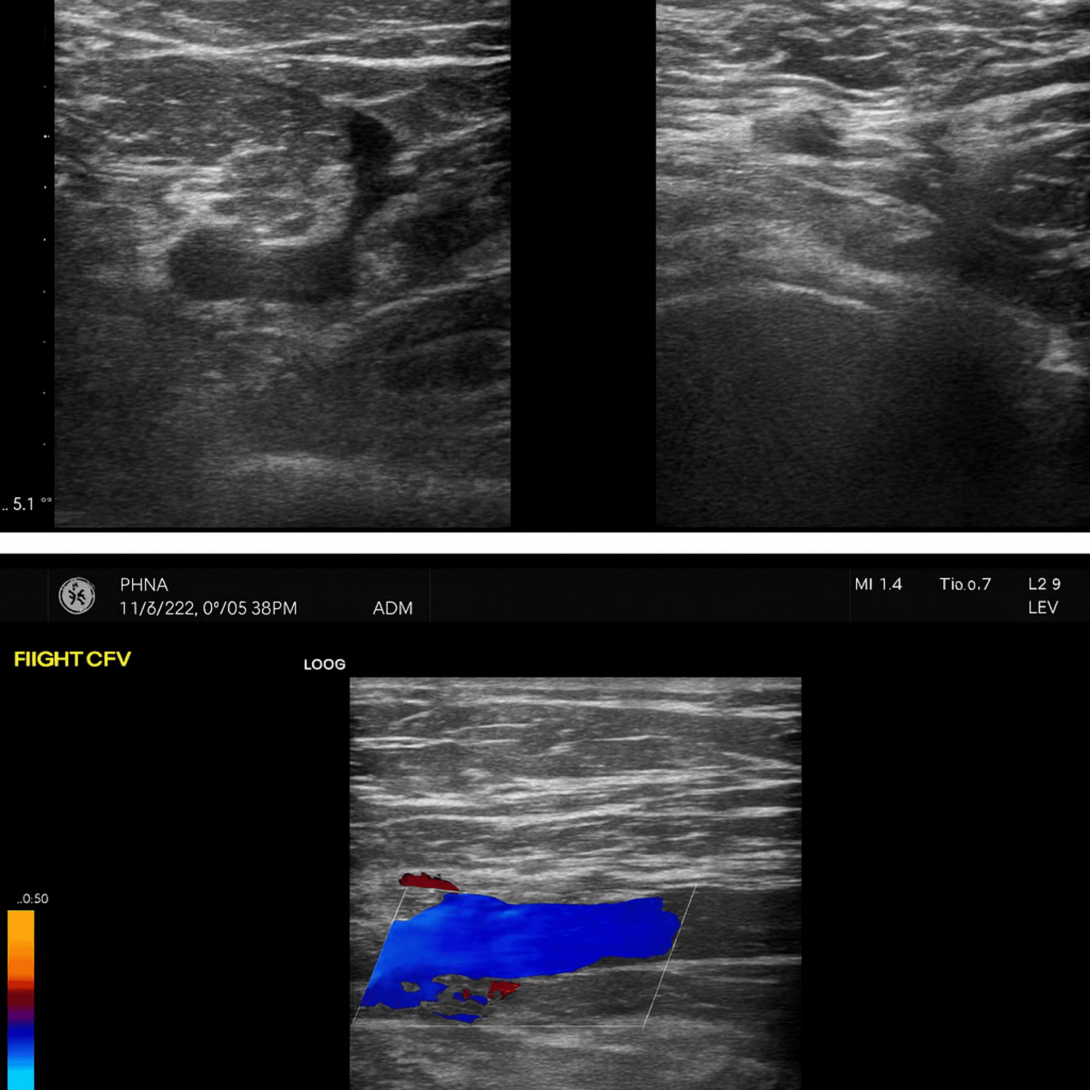
A. Deep venous thrombosis involving the left distal superficial femoral vein

**Figure 2:**
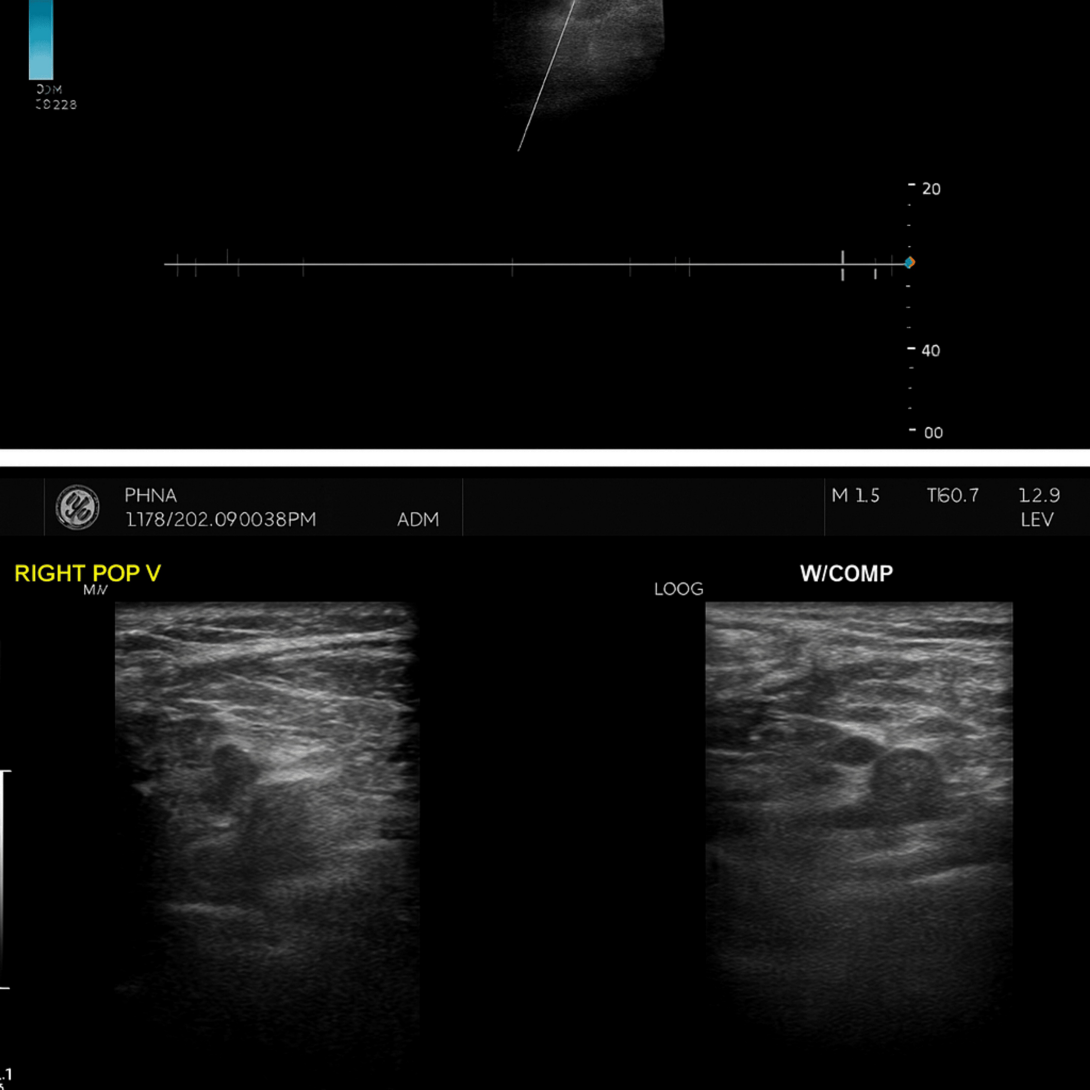
B. Deep venous thrombosis involving the popliteal vein

Doppler ultrasound confirmed thrombosis of the distal superficial femoral and popliteal veins (Figures 1, 2).

Autoimmune and thrombophilia screening, including prothrombin time (PT), international normalized ratio (INR), activated partial thromboplastin time (APTT), cardiolipin immunoglobulinG/ immunoglobulinM (IgG/IgM), and β2-glycoprotein antibodies, antinuclear antibody (ANA), and double‑stranded DNA antibody (DsDNA), was unremarkable (Table 2).

**Table 2:** Autoimmune and Thrombophilia investigations PT: prothrombin time; INR: international normalized ratio; APTT: activated partial thromboplastin time; IgG: immunoglobulinG; IgM: immunoglobulinM, DsDNA: double‑stranded DNA antibody; ANA: antinuclear antibody.

Laboratory Test	Results	Normal range
PT	14.7 s	9.5–12.5 s
INR	1.07	0.87–1.15
APTT	25.1 s	22.2–34.2 s
Cardiolipin IgG	1.72 CU	≤ 20 CU
Cardiolipin IgM	6.11 CU	≤ 20 CU
B2 Glycoprotein IgG	3.77 CU	≤ 20 CU
B2 Glycoprotein IgM	6.11 CU	≤ 20 CU
dsDNA (IFA)	Negative	Negative
ANA quantitative	Negative	Negative
C3	72 mg/dl	79–152 mg/dl
C4	31 mg/dl	16–38 mg/dl

The patient was initially started on therapeutic low molecular weight heparin (enoxaparin 1 mg/kg twice daily) and transitioned to oral apixaban (10 mg twice daily for seven days, followed by 5 mg twice daily) with a planned treatment course of three months. Following stabilization, he was discharged with arrangements for outpatient follow-up.

## Discussion

GLP-1RAs, including semaglutide, have become a cornerstone in the management of diabetes and obesity due to their favorable metabolic and cardiovascular profiles. However, concerns have emerged regarding their thromboembolic safety. Post-marketing surveillance and case reports have described events such as pulmonary embolism and atypical thrombosis in patients on semaglutide or liraglutide [4]. A recent report described portal vein thrombosis in a patient with a JAK2 mutation on semaglutide, suggesting the drug may trigger VTE in individuals already prone to thrombosis [6].

The mechanisms by which GLP-1RAs might predispose to VTE remain unclear. One possibility is hemoconcentration secondary to dehydration, which could lead to hypercoagulability and increase the risk of developing a VTE [7]. A previous case report described a patient who developed a VTE secondary to gastroenteritis, after several days of vomiting and diarrhea requiring an emergency department (ED) visit. Even in the absence of overt dehydration, reduced oral intake and subtle volume contraction may increase blood viscosity and clotting potential [8].

Lifestyle factors may also contribute to thromboembolic risk. Our patient reported prolonged sedentary behavior, which independently increases VTE risk, and a BMI in the overweight range, which may further synergize with drug-related effects [9].

Notably, his thrombophilia screen was negative. He had mild conventional risk factors, including overweight BMI (26.3 kg/m²) and prolonged sedentary behavior, which may have contributed to thrombotic risk. The relationship between semaglutide initiation and symptom onset strengthens the argument for a drug-associated event [10].

To date, there are no published reports of limb DVT specifically associated with oral semaglutide, making this case unique. While this isolated case suggests a possible association rather than a confirmed causal relationship, physicians should be cautious when GLP-1RAs are used off-label in adolescents, where baseline thrombotic risks may differ. When GLP-1RAs are used off-label for weight reduction in adolescents, where baseline thrombotic risks may differ from those of adult populations, unsupervised use may delay recognition of adverse effects.

## Conclusions

This case suggests a possible association between oral semaglutide and DVT in an adolescent with mild conventional risk factors [overweight BMI, sedentary behavior]. While rare, such complications highlight the importance of medical supervision, particularly in adolescents using GLP-1RAs off-label for weight management. Clinicians should maintain vigilance for thrombotic events in patients presenting with limb pain and swelling after starting semaglutide, especially in unsupervised, off-label use. Further studies are needed to clarify causality and establish safety profiles in pediatric and adolescent populations.
